# COVID 19 infection clinical features in pediatric patients in Southwestern Iran: a cross-sectional, multi-center study

**DOI:** 10.1186/s12879-023-08720-z

**Published:** 2023-11-25

**Authors:** Marzieh Jamalidoust, Mohsen Jalil, Zahra Ashkan, Moslem Sharifi, Rouhollah Hemmati, Anahita Sanaei Dashti, Mohammad Rahim Kadivar, Gholamreza Pouladfar, Ali Amanati, Seyeheh Sedigheh Hamzavi, Sadaf Asaie, Maryam Eskandari, Nasrin Aliabadi, Mazyar Ziyaeyan

**Affiliations:** 1 Clinical Microbiology Research Center, Shiraz University of Medical Sciences, Shiraz, Iran; 2 Emergency Medical Service, Yasouj University of Medical Sciences, Yasouj, Iran; 3 Department of Biology, Faculty of Basic Science, Shahrekord University, Shahrekord, Iran; 4 Departments of Pediatrics, Shiraz University of Medical Sciences, Shiraz, Iran

**Keywords:** COVID 19 Disease, Real-time PCR, Children, Symptoms, Underlying Disease

## Abstract

With the SARS-CoV-2 pandemic, the impact of recent coronavirus, especially in children, cannot be ignored. In this study, we evaluated the SARS-CoV-2 infection rates and associated features in children less than 18 years of age in “Fars” and “Kohgiluyeh and Boyer Ahmad”, provinces, Iran. 5943 children who were suspected cases to SARS-CoV-2 infection were enrolled in this study. Demographic and clinical data of SARS-CoV-2 patients were collected from 16 February 2020 to 20 June 2021. Underlying conditions were considered in this study as well. Among 5943 patients suspected COVID 19 cases, 13.51% were confirmed by real-time PCR assay. The female/male ratio was 1:1.3 with a mean age of 5.71 years. 11.2% of confirmed patients were transferred and admitted in Pediatric ICU. COVID 19 was significantly higher in children with malignancy and diabetes rather than those with other underlying diseases. Children of all ages were susceptible to COVID 19, and there is no significant difference between both sexes. Most of the COVID 19 cases were in 10–18 years old group. Among a number of children with different underlying diseases, children with malignancy had the highest rate of SARS-CoV-2 infection, followed by those with diabetes.

## Introduction

Coronaviruses (CoVs) are a large family of viruses that were first discovered in the 1960s. They are the largest group of viruses that cause respiratory and gastrointestinal infections. To date, four genera of coronaviruses (α, β, γ, and δ) have been identified as human coronaviruses, in either α or β genera [1]. SARS-CoV-2 or severe acute respiratory syndrome coronavirus-2 is the 7th family of *coronaviridae* which are capable of causing severe diseases in humans. This is a unique strain of RNA virus that has never been observed in humans. In addition to SARS-CoV-2, SARS and MERS viruses have emerged in Southern China (2002) and Saudi Arabia (2012), respectively, and have spread epidemiologically in the human population [2, 3]. HCoV-229E, HCoV-OC43, HCoV-NL63, and HCoV-HKU1 are the other coronaviruses that often lead to a common self-limited upper respiratory tract illness.

The first case of COVID 19 in Iran was reported in Qom on February 7, 2020. Meanwhile, WHO declared SARS-CoV-2 outbreak as a pandemic on March 11, 2020 [4]. At the time of writing, vaccination had been done in the vast majority of the population over the age of 12; more than 6.4 million people in Iran had been officially infected and about 132,000 (2%) of them had died.

SARS-CoV-2 infects people of all ages and, generally, the risk of developing a serious illness increases with age, especially in people over 60 years of age. Whether children are less likely to be infected with SARS-CoV-2 is an ongoing debate. However, several studies show differences in the COVID 19 severity, hospitalization and mortality rate at different ages. Generally, unlike other respiratory viruses such as RSV and Parainfluenza infections, children have milder symptoms when infected with SARS-CoV-2 infection.

Two cohort studies in China and the United States reported COVID 19 infection in 2% of Chinese and 1.7% of American children. The duration of viral shedding in children is up to 24 days from the onset of symptoms [5]. Most children infected with SARS-CoV-2 are asymptomatic or have mild symptoms, usually fever, cough, sore throat, gastrointestinal symptoms, and changes in smell or taste. A few articles reported that children infected with SARS-CoV-2 had symptoms similar to Kawasaki’s disease [6].

Over time, with the introduction of new variants of the SARS virus, the prevalence of the virus in children changed. For example, studies have shown that with the emergence of the delta strain, children’s admissions to hospital and more serious diseases significantly increases. The Omicron strain, which has emerged as a variant of concern or VOC should also has affected the children.

It is important to register and document the COVID 19 clinical presentations in children and young people to more rapid diagnosis and prompt management. In this study, we aimed to clarify the prevalence of COVID 19 among children less than 18 years of age in southwestern Iran and assess them during the 17 months of pandemic onset.

## Methods and material

### Data collection

In this non-interventional, uncontrolled, open, national, multicenter, cross-sectional study, we considered 5943 patients from newborns to 18 years of age, from 16 to 2020 to 20 June 2021 who were suspected or had clinically confirmed SARS-CoV-2 infection. The study centers were 11 university hospitals for children, a large number of health centers, and private offices of pediatricians, where children with suspected SARS-CoV-2 have been tested as outpatients in FARS, and 7 hospitals in “Kohgiluyeh and Boyer Ahmad” or K.B province.

All participants’ legal guardians provided written informed consent and the study was conducted in accordance with the guidelines of the declaration of Helsinki.

Participants were qualified for enrollment after they met the inclusion criteria including: (1) child (male or female) aged 1 day to 18 years, (2) consents of the caregivers of the participants (3) residency in Fars and K.B provinces.

The present study applied the Strengthening the Reporting of Observational Studies in Epidemiology (STROBE) statement (https://www.strobe-statement.org). The criteria for hospitalization were as follows:

- Presence of respiratory tract complaints; cough, sore throat, coryza (nasal stuffiness, rhinorrhea, repeated sneezing), difficult respiration of any type.
- Fever; body temperature ≥ 38 degrees c, with or without chills.
- Percutaneous oxygen saturation < 96%.
- A history of contact with a patient diagnosed with COVID 19.
-  A patchy ground-glass pattern on chest computed tomography images.

Demographic characteristics, initial symptoms and clinical signs, and disease severity were recorded.

### Sampling and nucleic acid amplification

Sterile Dacron swabs were used to obtain nasopharyngeal fluid (NPS) samples from patients. The collected specimens were kept on ice and transported immediately to the laboratory for detecting nucleic acids of SARS-CoV-2. Testing assays were performed using real-time PCR within 1–2 h following collection, as described by [7]. The patients’ data were obtained from hospital information system (HIS), the patients’ charts and real-time PCR assay results excel form.

### Statistical analysis

In this study, we used descriptive statistics to classify the study group based on SARS-CoV-2 PCR results. Numbers and percentages were used to report the classified variables. There was no missing data. The Chi-square test was employed to determine a possible relationship between the qualitative variables and the quantitative data. A significance level was considered as a P value ≤ 0.05. Symptoms related to PCR positivity among the patients were assessed using univariate and multivariate logistic regression (In the pre-selection stage, variables with a Pvalue < 0.20 in the univariable analysis were considered). The data were analyzed using SPSS software version 26. All available data were used to determine the positive predictive values ​​(PPV) and negative predictive values ​​(NPV) of each sign reported by the participants or their parents or guardian using MedCalc software.

This study was approved by the Ethics Committee of Shiraz University of Medical Sciences, Shiraz, Iran (IR.SUMS.REC.1401.006).

## Results

A total of 5943 patients were enrolled in the study, consisting of 3338 (56.2%) male and 2605 (43.8%) female. The samples were collected from all the main pediatric hospitals of 2 adjacent provinces in the southwest of Iran, during 17 months post SARS-CoV-2 pandemic. It is notable that in Fars province, 83.1% of patients were tested once, while 8.5%, 3.7%, 2.8%, 1.2%, and 0.7% of the infected children were tested 2–6 times, respectively; however, in K.B province, 93.7% of children were tested once and 6.3% twice. Their mean age was 5.71 (SD ± 5.75) years, the youngest and oldest being 1 day and 18 years old, respectively. Demographic characteristics of these participants summarized in Table 1. As shown in the Tables 1 and 803 (13.5%) of suspected children were affected by SARS-CoV-2 infection, with a mean age of 6.29 years (SD ± 6.04) (95% CI 7.5–14.7); 10.12% in Fars and 21.87% in K.B province.

**Table 1:** Demographic data of Covid-19 infection among suspected and infected Iranian pediatric patients

	Fars province (n = 4229)	K.B Province (n = 1714)	All patients (n = 5943)	SARS-CoV-2 infection (n = 803)	P-Value
Characteristics					
Age groups (years) 0–1 months 1 mounth-2 years 2–5 years 5–10 years 10–18 years	… 769 (18.2%) 1036 (24.5%) 580 (13.7%) 687 (16.2%) 1157 (27.4%)	… 269 (15.7%) 616 (35.9%) 329 (19.2%) 224 (13.1%) 276 (16.1%)	… 1038 (17.5%) 1652 (27.8%) 909 (15.3%) 911 (15.3%) 1433 (24.1%)	… 138 (17.2%) 202 (25.2%) 120 (14.9%) 106 (13.2%) 237 (29.5%)	
Sex Male Female	… 2396 (56.7%) 1833 (43.3%)	… 942 (55%) 772 (45%)	… 3338 (56.2%) 2605 (43.8%)	… 432 (53.8%) 371 (46.2%)	0.078
Comorbidity	73 (1.73%)	8 (0.46%)	81 (1.36%)	18 (2.2%)	

The biggest group that participated in this study was 1 month-2 years old (27.8%, n: 1651), followed by 10–18 years (24.1%, n: 1433), 1 day-1month (17.5%, n: 1039), 2–5 years (15.3%, n: 909), and 5–10 years (15.3%, n: 911).

Totally, the prevalence of SARS-CoV-2 PCR positive patients among 1 day-1 month, 1 m-2 years, 2–5 years, 5–10 years, and 10–18 years were 17.2%, 25.2%, 14.9%, 13.2%, and 29.5% respectively.

**Fig. 1:**
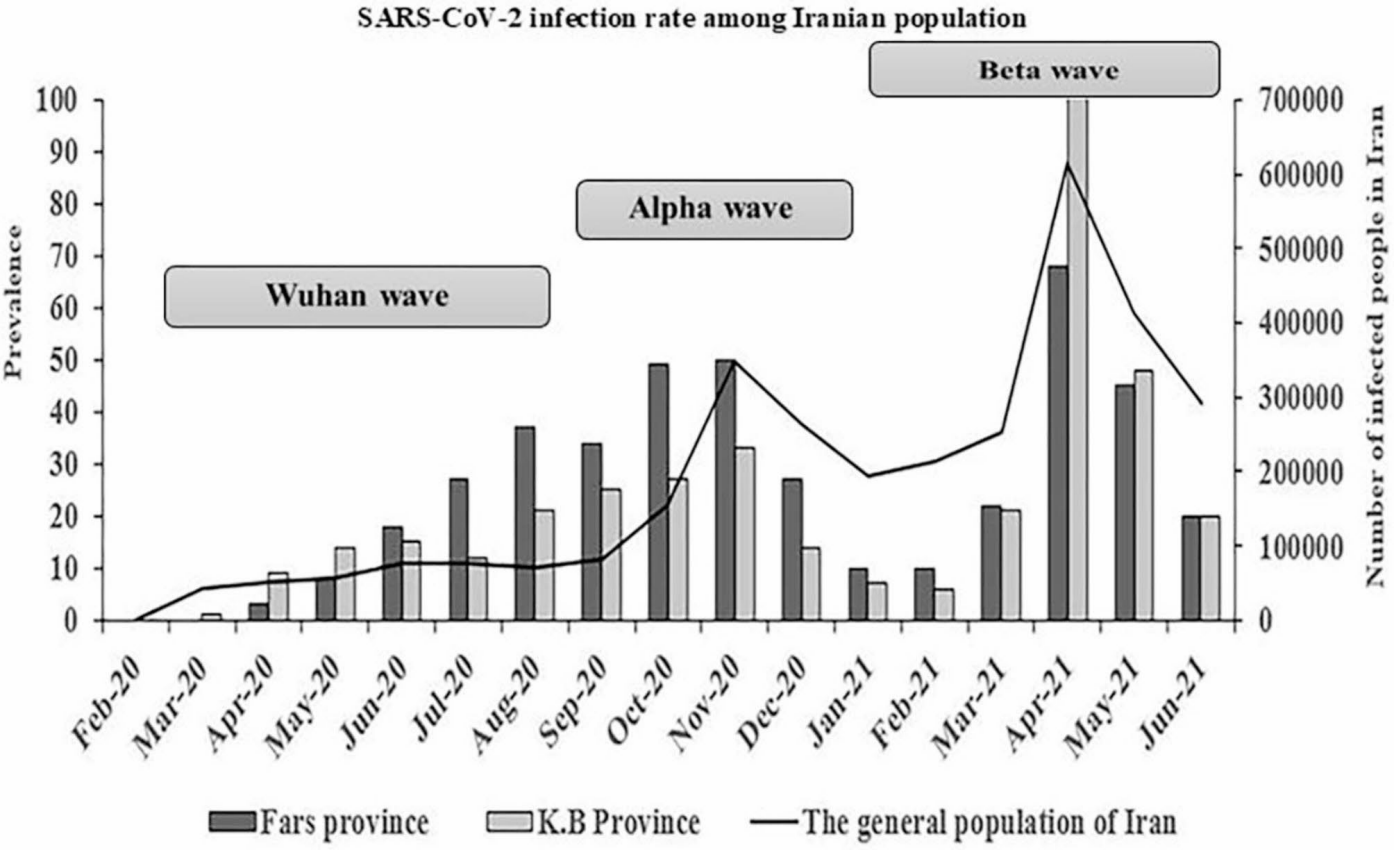
The infection rates among children during different months in the southwest of Iran

As shown in Fig. 1, there were 3 peaks in the prevalence of SARS-CoV-2 among children during the 1st of March 2020 to 20th of June 2021 that conform to general population.

**Fig. 2:**
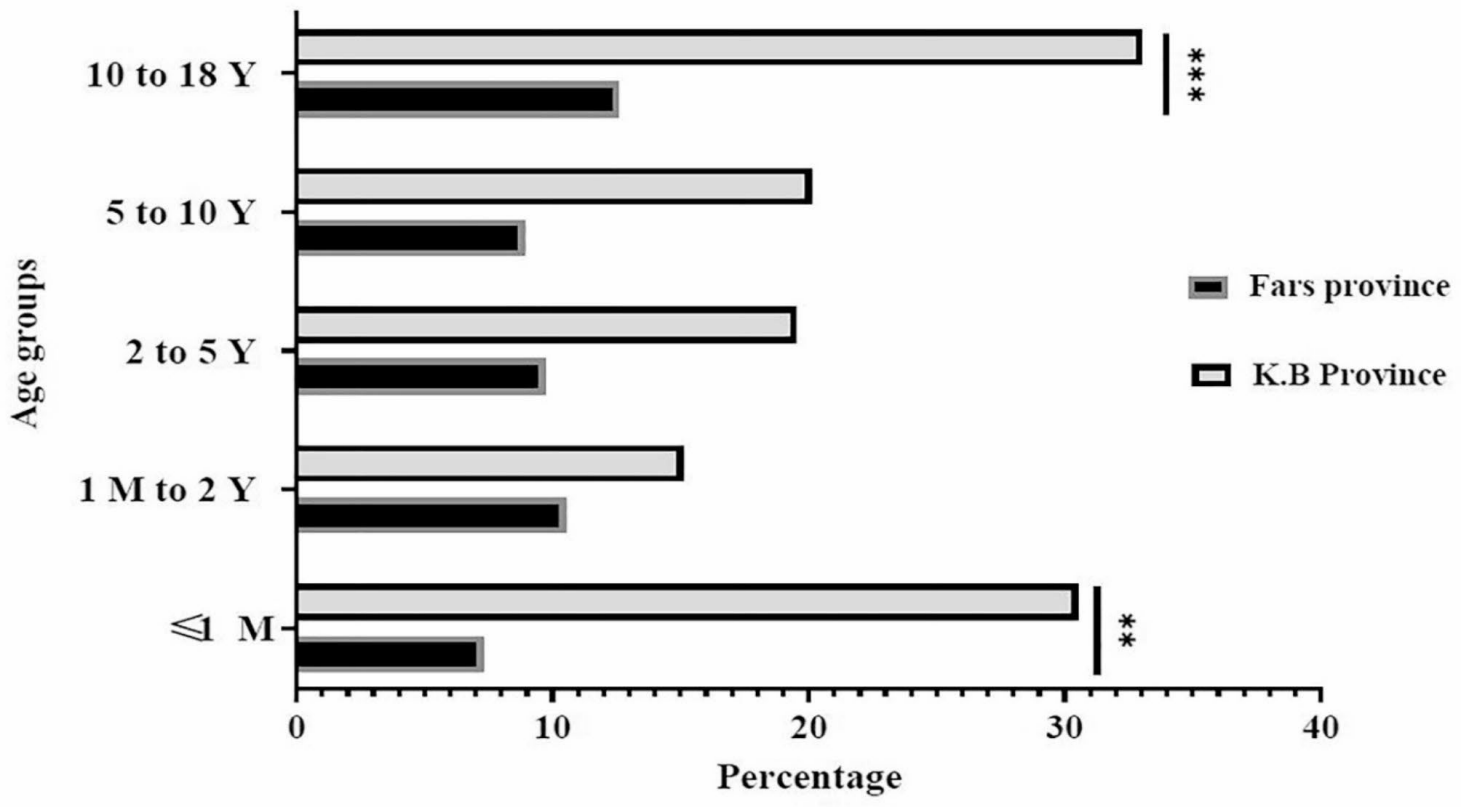
Prevalence of SARS-CoV-2 infection among the different age groups of children in Fars and K.B, southwest of Iran

As demonstrated in Fig. 2, the prevalence of COVID 19 in the 10–18 year old group and ≤ 1 month was significantly higher in K.B rather than Fars province with P-values of < 0.001 and < 0 0.01, respectively.

Clinical symptoms in PCR positive and PCR negative patients were compared (Table 2). Generally, fever was the most common symptom in both groups it was significantly higher in PCR positive ones with a P value of ≤ 001.

**Table 2:** Symptoms of patients suspicious to and affected with COVID 19, the southwest of Iran, from February 2020 to June 2021

Symptoms	Number of individuals testing positive via SARS-CoV-2 PCR N (%)	Number of individuals testing negative via SARS-CoV-2 PCR N (%)	Parameters (%)			
			Sensitivity	Specificity	PPV	NPV
Fever	446(55.5%)	2437(47.4%)	29.15	72.83	57.29	45.14
Cough	224(27.9%)	747(14.5%)	15.18	82.29	24.90	71.48
Runny nose	5(0.62%)	33(0.64%)	100	0.93	1.54	100
Shortness of breath	67(8.3%)	454(8.8%)	14.29	86.33	15.55	85.11
pneumonia	3(0.4%)	15(0.3%)	100	1.23	0.61	100
Nausea	66(8.2%)	414(8.05%)	98.48	3.80	8.39	96.51
Diarrhea	120(14.9%)	789(15.3)	11.67	88.87	15.55	85.13
Abdominal pain	20(2.5%)	206(4%)	100	1.02	2.51	100
Anorexia	42(5.2%)	130(2.5%)	100	6.33	9.76	100
Chest pain	13(1.6%)	31(0.6%)	0	0		
Headache	65(8.1%)	141(2.74%)	100	0.81	8.16	100
Dizziness	26(3.2%)	138(2.7%)	7.69	97.17	8.34	96.91
Myalgia	79(9.8%)	102(2%)	100	0.97	9.92	100
Fatigue	18(2.2%)	106(2.1%)	100	0.32	4.29	100
General weakness	39(4.85%)	204(4%)	100	4.38	9.54	100

**PPV**: Positive Predictive Value, **NPV**: Negative Predictive Value

In addition, cough, sore throat, shortness of breath, respiratory symptoms, anosmia, loss of taste, abdominal pain, chest pain, headache, myalgia and general weakness were the other symptom that were significantly higher in PCR positive patients.

The multiple regression analysis followed by univariate analysis revealed that fever (p value = 0.007), cough (p value = 0.000), sore throat (p value = 0.006) and general weakness (p value = 0.025) were the most prevalent clinical manifestations of COVID 19 affected children. In addition the rate of patients who had received intensive care and mechanical ventilation were (6.18% and 19.98%) and (3.8% and 11.2%) in K.B and Fars provinces, respectively.

25% of children with direct contact with patients with COVID 19, had positive tests.

**Fig. 3:**
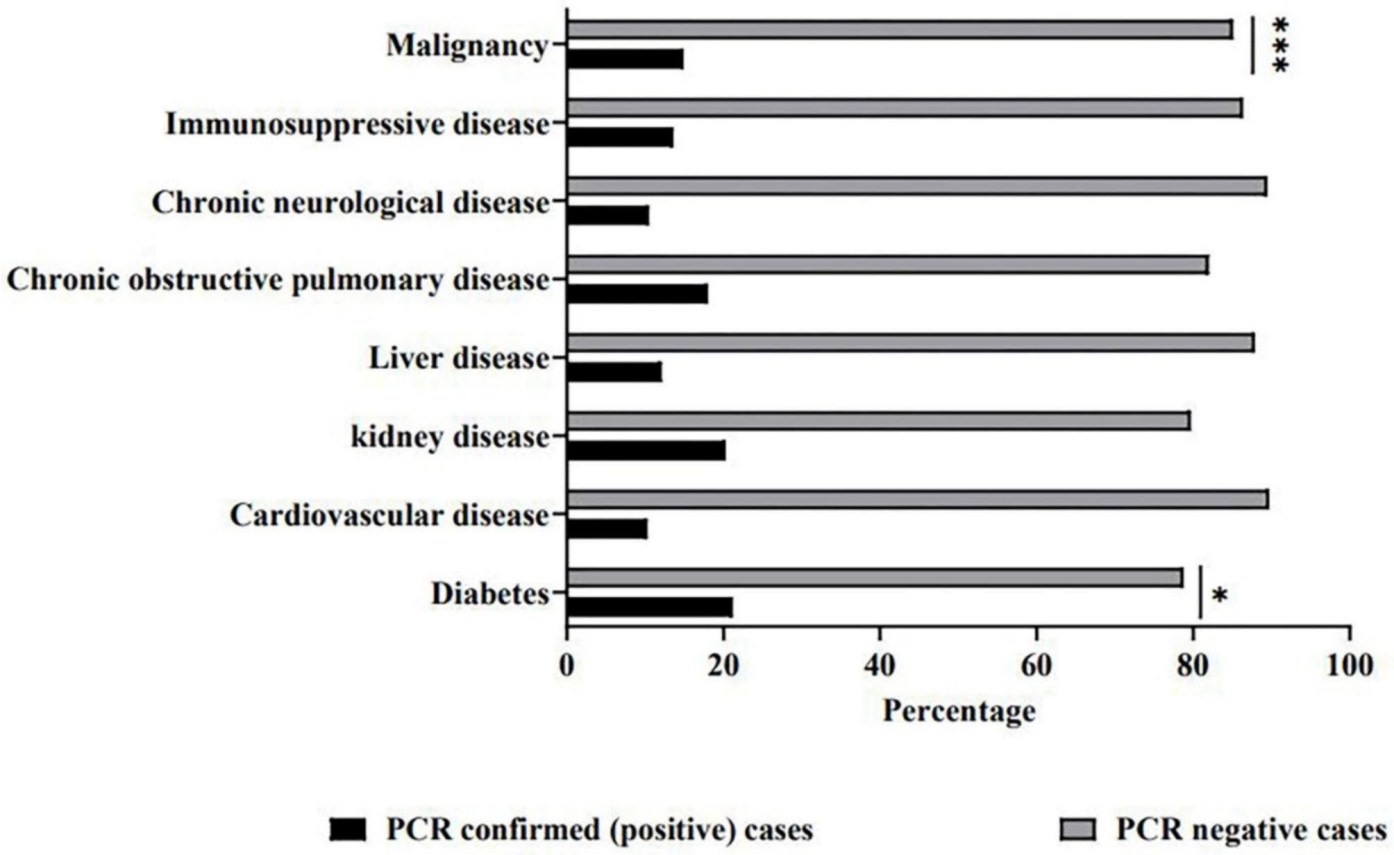
Prevalence of SARS-CoV-2 infection among Iranian children with underlying condition from February 2020 to June 2021

This study evaluated the prevalence of COVID 19 in children with different type of co-morbidity. Totally 23.5% of suspected patients were immunocompromised. As shown in Table 1, malignancy (7.7%) was the most prevalent underlying disease followed by chronic neurological disease (3.9%), cardiovascular disease (3.4%), liver disease (2.9%), chronic pulmonary disease (1.5%), diabetes (1.4%), Immunosuppressive diseases (1.4%), and kidney disease (1.3%). Statistical analysis demonstrated that SARS-CoV-2 infection was significantly higher among children with malignancy (p ≤ 0.001) or diabetes (p ≤ 0.05) (Fig. 3).

## Discussion

This study assessed the presence of SARS-CoV-2 infection rate using Taq man real-time PCR assay and the epidemiological and clinical data of them, in the southwest of Iran during 17 months of post-pandemic period. The mean age of infected patients was 5.71 years that is consistent with Dong et al.’s study [8]. Global data shows mixed gender distribution of COVID 19, and in our study a slight male predominance was noted (56.2% vs. 44.8%).

13.5% of the suspected patients were confirmed COVID 19 cases, based on positive PCR. The SARS-CoV-2 positive rate in K.B province is higher than in Fars province (21.87% and 10.12%, respectively). The higher rate of COVID 19 infection in K.B province may be due to the particular cultural conditions; more social and interfamilial contacts and large funerals in this particular geographic area. On the other hand, unlike the Fars province, where 20% of the enrolled population was outpatient, K.B province enrolled patients were totally inpatients. Totally, our prevalence was very similar to Hosseininasab et al.’s study in Kerman province of Iran (13.4%). COVID 19 affected children (69%) were more prevalent on admission screening, in the hospital of Karachi in Pakistan [9], unlike our study. The prevalence of SARS-CoV-2 infection in males and females (14% vs. 13%, respectively) was similar and consistent with that of Min Jin’s study [10].

The highest numbers of SARS-CoV-2 suspected children were in the 1 month to 2 year age group, but the most affected children were between 10 and 18 years old (P = 0.003). This high rate of COVID 19 –like respiratory infections in the 1 month to 2 year age group is predictable due to lower efficacy of the immune system. The other factor may be high referral rate of this young children to the health care centers due to their susceptibility. The higher prevalence of COVID 19, in 10–18 years old age, may be due to a number of reasons including increased expression and affinity of ACE-2 receptors that facilitate viral entry [11], decreased pre-existing immunity [12], more social interactions, higher risk of CMV co-infection in older age [13] etc. [14] [15].

According to Dong et al. report more than 90% of COVID 19 affected children were asymptomatic or with mild to moderate symptoms [8]. Other studies in Egypt, China, and United States reported that symptomatic children showed mild symptoms and did not have severe disease or deaths [8, 16, 17].

In consistent with ours’ cough, respiratory complaints, headache, and myalgia were significantly more common in SARS-CoV-2 positive children, in other studies. [18].

In Zhang et al.’s study on Chinese SARS-CoV-2 infected children, the most common symptoms were fever (76%) and cough (62%) [19]. Fever (50%) was the most common clinical characteristic among children with COVID 19 in Karachi, Pakistan, too [9, 20].

Notably and fortunately, the symptoms of those testing negative (having COVID 19 like complaints), were recorded in our study, enabling to measure the sensitivity, specificity, PPV, and NPV of individual symptoms.

Gastrointestinal complaints such as diarrhea, nausea, and abdominal pain were observed in our patients like other studies [19, 21, 22].

The study performed by Parri et al. showed gastrointestinal symptoms in Italian children [23]. Diarrhea, nausea, and abdominal pain were present in children infected with SARS-CoV-2 in Northern Iran [18]. Gastrointestinal complaints were seen in 25% of children in Karachi, Pakistan [9]. Gholami et al. in a systematic review and meta-analysis reported that the percentage of vomiting (10%) and diarrhea (5%) was not very high [24]. A meta-analysis study by Akobeng reported the prevalence of vomiting was 10.3% and diarrhea 12.4%, and generally 22.8% of children had gastrointestinal symptoms [25].

Neurologic complaints including headaches and confusion were reported by some of the children in this study. Also, these neurologic complaints were present in the significant number of children in Northern Iran in the study of Shahbaznejad, et al. [18]. Hong, et al. and Zimmermann and Curtis reported neurologic complications in pediatric COVID 19. However, in Pakistan, neurological complications were low (5%). The common symptoms among children with neurologic complaints were seizures and neuromuscular weakness [9].

In the present study, 26.16% of the patients were transferred to the ICU. In Europe, the rate of PICU admission was 8% [26]. The PICU admission rate in New York City in 46 hospitals was 28% [27]. In another study in Northern Iran, the patients admitted to PICU were 20% [18]. Tagarro et al. reported that among confirmed cases only 16% were transferred to PICU in Spain [28]. However, in children with COVID 19, the exact rate of PICU admission is still unknown. Different PICU admission policies can be the reason of these different results [18]. Also, all articles reveal that, according to the existing knowledge, the severity of disease in children is much less than adults and its cause is unknown [29, 30].

Several studies have also reported an association between comorbidities and COVID 19 infection [26, 31]. Cardiac and circulatory congenital anomalies and type 1 diabetes were the strongest risk factors for severe COVID 19 [32, 33].In our study, pediatric comorbidities included malignancy, chronic neurological disease, cardiovascular disease, liver disease, chronic pulmonary diseases, diabetes, immunosuppressive disease, and kidney disease. SARS-CoV-2 infection is significantly higher in children with cancer and diabetes than in those with other underlying diseases. A study of Omigi et al. showed underlying diseases in younger age, particularly in pre-school aged children, are predictors of disease severity [34–36].

Different studies showed that most children with COVID 19 got infected through contact with infected patients or family cluster; 98.69% [29]. All children in Baki’s study had been infected by contact with confirmed COVID 19 family members [16]. Some studies in China reported similar results [37–40] but in our study only 25.3% of the infected children had positive contact.

Our research showed 5.8% of PCR positive children had a history of recent travel, while some studies revealed a lower rate (0.089%) [29].

In conclusion, 13% of suspected children to COVID 19, confirmed to be affected SARS-CoV-2 infection using real-time PCR assay and 26% of them were admitted to PICU. SARS-CoV-2 infection is significantly higher in children with cancer and diabetes than in those with other underlying diseases. Fever, cough, dyspnea and other respiratory symptoms, headache, and myalgia were significantly more common in SARS-CoV-2 positive children rather than negative children.

## Data Availability

The data that support the findings of this study are available on request from the corresponding author.
